# Compassionate faces: Evidence for distinctive facial expressions associated with specific prosocial motivations

**DOI:** 10.1371/journal.pone.0210283

**Published:** 2019-01-23

**Authors:** Caroline J. Falconer, Janek S. Lobmaier, Marina Christoforou, Sunjeev K. Kamboj, John A. King, Paul Gilbert, Chris R. Brewin

**Affiliations:** 1 Department of Clinical, Educational and Health Psychology, University College London, London, United Kingdom; 2 Institute of Psychology, University of Bern, Bern, Switzerland; 3 Mental Health Research Unit, University of Derby, Derby, United Kingdom; University of Belgrade, SERBIA

## Abstract

Compassion is a complex cognitive, emotional and behavioural process that has important real-world consequences for the self and others. Considering this, it is important to understand how compassion is communicated. The current research investigated the expression and perception of compassion via the face. We generated exemplar images of two compassionate facial expressions induced from two mental imagery tasks with different compassionate motivations (Study 1). Our kind- and empathic compassion faces were perceived differently and the empathic-compassion expression was perceived as best depicting the general definition of compassion (Study 2). Our two composite faces differed in their perceived happiness, kindness, sadness, fear and concern, which speak to their underling motivation and emotional resonance. Finally, both faces were accurately discriminated when presented along a compassion continuum (Study 3). Our results demonstrate two perceptually and functionally distinct facial expressions of compassion, with potentially different consequences for the suffering of others.

## Introduction

Compassion is typically defined as a sensitivity to the suffering of the self and others with a desire and commitment to try to alleviate and prevent that suffering [1–3]. The compassion motive requires a range of competencies including empathy, distress tolerance, perspective taking, sympathy, and wise action. Our capacity for compassion is rooted in ancient motivational systems that evolved to protect and care for offspring [4, 5], but can now be extended to friends and even strangers [4, 6, 7]. There is growing evidence that the giving and receiving of compassion has profound effects on a range of physiological, psychological and social processes [8]. Given this, it is important to understand how compassion is successfully communicated and perceived. The purpose of the current research was to investigate how compassion is communicated via facial expressions and how this is perceived by others.

Facial expressions are the most important source of non-verbal information for humans interacting in social situations. There is extensive research investigating facial expressions of emotions such as happiness, anger, fear, disgust, and surprise, which are cross-culturally identifiable and regarded as universal. However, there has been little research into facial expressions corresponding to motivational states like compassion. Whether or not they turn out to be universal, information about the facial expressions corresponding to such states can be valuable in designing and tuning interactions that contain a compassionate element, such as therapeutic interventions [9].

What might compassionate facial expressions look like? As outlined above, compassion is a process of (a) identifying the suffering of the self and others that is then (b) accompanied by a desire and commitment to alleviate and prevent that suffering. Part (a) might therefore involve a facial expression communicating a reflection (resonance) and understanding of suffering. Part (b) might be communicated with an open, unjudging expression with features denoting affiliation, approach behaviour and kindness (e.g. a gentle smile) or a worried expression denoting concern for another.

Goetz et al. [10] have recently reviewed the literature on compassion-related non-verbal displays. The early studies did not investigate compassion *per se* but vicarious displays of sympathy (“other-focused concern”) on the face [11–15]. Sympathy was coded from the faces of children and adults who were exposed to sympathy-inducing vignettes or videos. Facial expressions of sympathy were characterized by “*eyebrows pulled down flat and forward over the bridge of the nose, furrowing in the centre of the brow…, eyelids not pulled in tight or raised, head and body oriented forward, bottom eyelids sometimes raised slightly, and lower face relaxed” [11].* Interestingly, facial displays of sympathy were found to be positively associated with measures of empathy, sympathy, pro-social behaviour and heart rate [11, 13–15]. This is also consistent with more recent research [16] on the empathy-altruism hypothesis that stipulates a sensitivity to the emotional states of others in need drives empathic concern which in turn motivates prosocial behaviours [17, 18].

**Fig 1:**
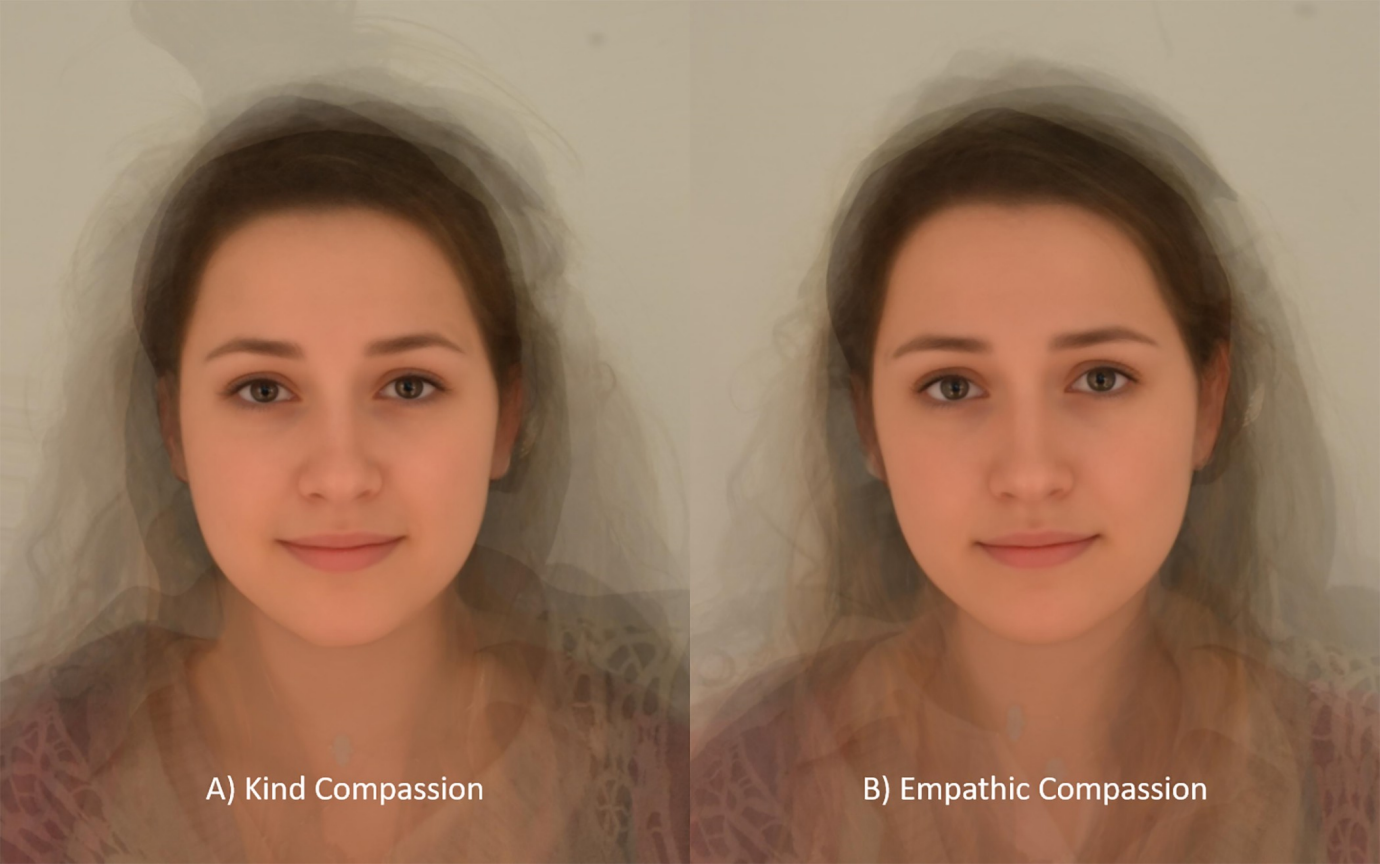
Prototypes of Kind (A) and Empathic (B) Compassion. The composite images are averages of the 10 faces that received the highest compassion ratings.

In two other studies, two male and two female actors were photographed expressing sympathy (i.e. with oblique eyebrows and a slight head tilt forward: Fig 1A). In a forced-choice task, naïve participants were required to identify a range of emotions including “sympathy” in an array of facial stimuli [19]. For the two male faces, 33% of participants correctly identified the facial displays of sympathy above chance, which was set at 7.5%. For the two female faces 42% of participants labelled the images as sympathy. However, 36% of responses to the female sympathy faces were categorised as “sadness” and 32% of responses to male faces were categorised as “no emotion”. In a second study, Haidt & Keltner [20] showed the same images to American and Indian students but changed the label from “sympathy” to “compassion” without any explanation. American students identified the “compassionate” faces more often than their Indian counterparts (30% vs. 17% of participants), but they more often rated them as showing sadness (37% of participants). The authors stated that labelling within the range of 20–40% may indicate that participants are guessing the expression from several possibilities.

In another recent study, McEwan et al. [21] set out to develop and validate a set of face stimuli depicting ‘kind-compassion’. The resulting images portrayed an ‘open face’ with a slight smile and the label ‘compassion/warmth’ was statistically the strongest attributed to the faces. However, close examination of the McEwan faces leads us to question whether this expression (particularly the soft smile) encompasses all aspects of the emotion, motivation and behaviour of individuals when they aim to comfort someone in distress. While expressions of friendliness, affiliation or kindness may communicate openness, non-judgement and approachability (all important elements for compassion), there may be a tendency to confuse kindness with happiness, which, in the presence of distress, may be experienced as invalidating and aversive to individuals [22, 23]. This concern is substantiated from research showing that smiling faces are less positively experienced when the viewer is in physical pain [24, 25].

The McEwan faces provide evidence of a reliable identification of expressions of kindness-focused compassion (part b), but with less of the associated sadness or emotional resonance (part a) detected in previous research that may be appropriate in some situations [26]. The current research therefore aimed to investigate expressions and perceptions of two different facial expressions of compassion that not only encompass competencies of sympathy and kindness but also empathy (emotional resonance and perspective taking). We term the former expression as ‘kind-compassion’ and the latter as ‘empathic-compassion’.

The decision to study static rather than moving facial expressions was taken despite the recognition that compassion is a dynamic inter- and intra-personal process. Given the lack of previous, systematic research into compassionate facial expressions, using static images allows us to isolate key components of compassion expression across our dataset. The face processing literature shows that dynamic facial expressions of emotion can be more readily recognised and produce greater levels of brain activity and perceived intensity of emotion [27, 28]. Nevertheless, if we can provide evidence of perceptual differences between our two types of static compassionate faces then this paves the way for further investigations using dynamic facial stimuli.

### Study aims

In Study 1 we used computer graphics software to generate a composite image of these two types of compassionate facial expressions, combining the responses of participants acting under two sets of mental imagery instructions. The use of composites ensures that differences are not attributable to the way specific individuals choose to express different emotions but are generalizable. We also explored whether the ability to express compassion on the face (as rated by others) was associated with higher self-report measures of compassion and empathy, which would be in line with previous research (11, 13, 14, 15, 16). In Study 2 we used sets of these composite images to assess the perception of compassion, asking participants to select which of the two faces best depicts a specific compassionate motivation (kind vs empathic). In Study 3 we used a discrimination paradigm to assess the ease of recognizing the two types of compassionate face when the faces were presented along a continuum from neutral to highly compassionate faces.

## Study 1

### Materials and methods

#### Participants

For the first part of Study 1, 32 women (mean age 20.97 years, SD = 2.29) were recruited to have their photographs taken. These are referred to as 'actors', although this does not denote their profession, but rather describes the activity they undertook as part of the study. We recruited only women because in a pilot phase we found it difficult to discern any differences in the photographs of male actors when comparing the two compassion conditions with a neutral expression (*see* Discussion). Eight actors were East-Asian, one was of mixed ethnicity and the remaining were white Caucasian. Participant numbers were gauged based on previous research in the field: studies generating composites have used within the range of 28 to 60 individuals posing for photographs [29–31].

In the second part of Study 1, 70 participants (60 females; mean age 20.69, SD = 5.58) were recruited to rate the photographs. These ratings were then used in correlation analyses and a power calculation indicated that 68 participants were required to detect a medium effect size (r = .30) with an alpha of .05 (1-tailed) and 80% power. Participants across all studies provided written informed consent to take part. Participants were given either academic credit or a small monetary payment for their participation. Ethical approval for all studies was obtained from the ethics committee of the Research Department of Clinical, Educational and Health Psychology, University College London.

#### Self-report measures

The Interpersonal Reactivity Index (IRI) [32] was used to measure two components of empathy. The subscales used in this study were Perspective Taking and Empathic Concern, assessing cognitive and affective empathy respectively. Participants had to respond to 7 statements for the Perspective Taking scale (e.g. “I try to look at everybody’s side of a disagreement before I make a decision”) and the Empathic Concern scale (e.g. “I often have tender, concerned feelings for people less fortunate than me”). Participants rated on a 5-point Likert scale how well each statement described them (0 = “Does not describe me well”; 4 = “Describes me very well”). Interrater-reliability of these scales has been shown to be sufficiently high, with Cronbach's alphas in a female sample 0.62 and 0.70 for Perspective Taking and Empathic Concern, respectively.

The Compassionate Love Scale for close others [33] is a measure of compassion for others. This scale consists of 21 items that assess compassionate love with the target specified as close others. Participants responded to statements (e.g. “It is easy for me to feel the pain and joy experienced by my loved ones”) on a 7-point Likert scale (1 = “Not true of me”; 7 = “Very true of me”). Cronbach’s alpha was 0.90 in a sample of university students [34].

#### Procedure

In the first part of Study 1, colour photographs of actors were taken with a Nikon 3100 DLSR camera that was positioned approx. 1.5 meters from where the actor sat in a white, light restricted room. Photographs of neutral expressions were taken first, with no specific instructions other than to relax. For the compassion expressions actors first read standardised instructions. They then listened to audio recordings of the instructions for empathic- and kind-compassion. The photograph was taken after the audio ended and participants indicated they were ready by looking into the camera. Practice trials were allowed until the actor felt satisfied that their facial expression corresponded to the motivation described in the instructions. Each actor provided three images: neutral, kind-compassion and empathic compassion. The self-report questionnaires were then administered, with order randomised across actors.

Compassionate facial expression instructions required actors to recall an autobiographical event that involved them compassionately responding to another's suffering. These instructions aimed to revive feelings and motivations associated with those instances. The first set of instructions was concerned with capturing sympathy and the desire to be helpful/supportive through kindness ([4]; ‘kind compassion’). In addition to sympathy and kindness, instructions for the second display also incorporated empathy, such as emotional resonance and an understanding of the feelings and perspective of the person suffering (‘empathic compassion’).

The instructions actors received for kind compassion were similar to those used by McEwan et al. [21]:

“Try to recall a time when you have had to comfort someone you know well who was feeling very bad about something in their work or personal life. In this situation, you may have wanted to express your understanding of their situation. Replay this event in your mind’s eye and take a moment to recreate within you the compassionate feelings that you had towards that person and your wish to comfort them. You may have experienced feelings of kindness and gentleness towards this other person and wanted to reassure and soothe them. Once you have re-established these feelings try to express them with your face. If you are having difficulties try to imagine this person sitting in front of you now.”

For the expression of empathic compassion participants received the following instruction. The underlined areas are to highlight key differences between the kind and empathic instructions:

“Try to recall a time when you have had to comfort someone you know well who was feeling very bad about something in their work or personal life. In this situation, you may have wanted to express your understanding of their emotions and your shared emotional pain. Replay this event in your mind’s eye and take a moment to recreate the compassionate feelings that you had towards that person, your shared suffering and your wish to comfort them. You may have experienced feelings of deep concern and sympathy towards this other person. You may have experienced feelings of kindness and gentleness towards this other person and wanted to reassure and soothe them. Once you have established these feelings try to express them with your face. If you are having difficulties try to imagine this person sitting in front of you.”

In the second phase of the study, participants rated the 64 compassionate photographs via the online platform Qualtrics (Provo, Utah, USA). For each photograph, participants rated on a 7-point scale the extent to which five basic emotions (happiness, sadness, fear, anger, disgust) as well as compassion were being expressed on the face (1 = “Not at all”; 7 = "Very much so”). Participants were given the definition of compassion as outlined in the introduction. Participants were instructed to set their computer screens to a resolution of 1280 x 1024 prior to starting the study.

Faces in each of the instruction conditions (empathic or kind compassion) were sorted based on the average from the 7-point compassion rating scale across participants. To generate composite images of the compassionate faces, the 10 highest rated faces were averaged using the graphics software PsychoMorph [35]. Using 178 facial markers, PsychoMorph allows the shape and position of the eyes, brow, nose, mouth, cheeks, chin, ears and the outer structure of the face to be registered (for a more detailed description of prototype creation see Tiddeman et al. [35]. Averaging a set of faces with similar characteristics is likely to retain expressive features shared by the individual images. Likewise, the features that are not consistent across participants are averaged out. The averaged faces can therefore be regarded as prototypical expressions of each type of compassion.

### Results and discussion

The composite images of kind and empathic compassion can be seen in Fig 1A and 1B, respectively. The kind and empathic compassion prototypes were both made up of nine white Caucasians and one East-Asian. The average ratings of photographs that contribute to the compassion composites are displayed in Table 1. Dependent samples t-tests revealed that there were no statistical differences between the two groups of faces that make up the compassion composites on any of the emotions being expressed (p values < 0.1).

**Table 1:** Mean (SD) of top 10 rated faces for kind and empathic conditions.

	Mean (SD) Expression Rating					
Condition	Happiness	Sadness	Anger	Fear	Disgust	Compassion
Kind Compassion	3.84 (1.37)	1.95 (.90)	1.28 (.12)	1.55 (.49)	1.35 (.15)	3.59 (.22)
Empathic Compassion	2.79 (1.37)	2.50 (1.02)	1.38 (.15)	1.83 (.63)	1.48 (.23)	3.55 (.20)

A summary of the self-report empathy and compassion scores are presented in Table 2. We conducted Pearson correlations to examine the associations between the participant ratings of the photos in the two compassion conditions with the self-report measures from actors. Bonferroni corrections compensating for multiple tests would have resulted in setting alpha at .005, but were contra-indicated as the dependent variables were correlated [36]. We found that participants’ compassion ratings of actors expressing kind compassion were negatively associated with the actors’ perspective-taking self-report scores, r(30) = -.42, p = .02: Actors rated low for compassion by participants reported higher perspective-taking scores. A similar association was found for faces in the empathic compassion condition, although this was not statistically significant, r(30) = -.32, p = .07. Scatterplot inspection revealed no indication of any non-linear relationships between the two variables. There were no other significant correlations between trait measures and rated compassion.

**Table 2:** Correlations between self-report measures and rated expression of compassion.

	K-Comp	E-Comp	IRI-EC	IRI-PT	CFO
K-Comp	1	.83**	-.12	-.42*	-.26
E-Comp		1	-.27	-.32	-.17
IRI-EC			1	.28	.64**
IRI-PT				1	.34
CFO					1
*Average*	2.96	2.79	20.78	19.38	5.13
*SD*	.60	.62	4.24	4.46	1.12

NB: K-Comp = Kind Compassion; E-Comp = Empathic Compassion; IRI-EC = Empathic Concern scale of the Interpersonal Reactivity Index; IRI-PT = Perspective Taking scale of the Interpersonal Reactivity Index; CFO = Compassion for Others.

** p < 0.01

* p < 0.05.

The primary output from Study 1 was the generation of two facial compassion prototypes. These composite images were generated from two sets of mental imagery instructions. The kind compassion instruction focused on the compassionate qualities of sympathy, soothing and kindness when confronted with suffering. The empathic compassion instructions included the same as those in the kind compassion condition but with an additional focus on the shared experience of suffering (i.e. emotional resonance) and concern. The faces that made up the prototypes did not differ in terms of the rated expression of compassion or basic emotions, such as happiness and sadness, suggesting that at the individual level these differences are subtle and not easily discerned.

In light of previous research showing an association between sympathy, empathy, pro-social behaviour and vicarious facial expressions of sympathy [11, 13, 14], it would be reasonable to assume an association between compassion expression and trait levels of empathy and compassion. We found an association between kind compassion and perspective-taking. However, this was a negative correlation whereby participants with higher self-reported perspective-taking abilities scored lower on facial compassion. While there is research to suggest an association between empathy traits and compassion expressions, some of this research [16, 37] focuses on the expression of basic emotions and prosocial behaviour in the form of cooperation. The expression of compassion in this study is a more complex expression in response to the suffering of others and the disparity may be explained by higher-order beliefs about compassion. The perspective-taking component of empathy is associated with cognitive empathy and understanding the mental states of others [33, 38]. Our ability to be compassionate is affected by interpersonal concerns or fears of compassion such as “does this person actually want my help?” or “if I help them will they take advantage of me?” [39]. It could be the case that higher levels of perspective-taking abilities, albeit self-reported, resulted in participants second-guessing their compassionate motives and behaviour and, consequently, expressing less.

The overall absence of correlations between the actors’ compassion expressivity and their trait scores could also reflect a discrepancy between self-report measures and actual compassionate motivations and behaviours. The research field is dominated by self-report measures of compassion with few objective measures [40].

While there were no statistical differences in the averages of the top ten faces across compassion and various emotions, when they were averaged to make a composite there were discernible differences between the two faces (i.e. they were not identical). In light of this, the aim of Studies 2 and 3 is to determine whether there are any perceptual or conceptual differences between the two kinds of facial expressions of compassion which, despite being hard to discriminate at an individual level, are captured in the composites.

## Study 2

### Methods

#### Participants

Fifty-four participants (42 females; mean age 33.5, SD = 9.2) took part in this study. Participants had not previously participated in Study 1. Formal power analysis for the within-subject analyses was precluded by the absence of information about the likely correlation between the paired observations. Our sample size was therefore gauged from previous literature in the field; studies range from 24 to 50 participants for discrimination tasks [29–31, 41]. The results suggest sample size was adequate.

#### Procedure

**Fig 2:**
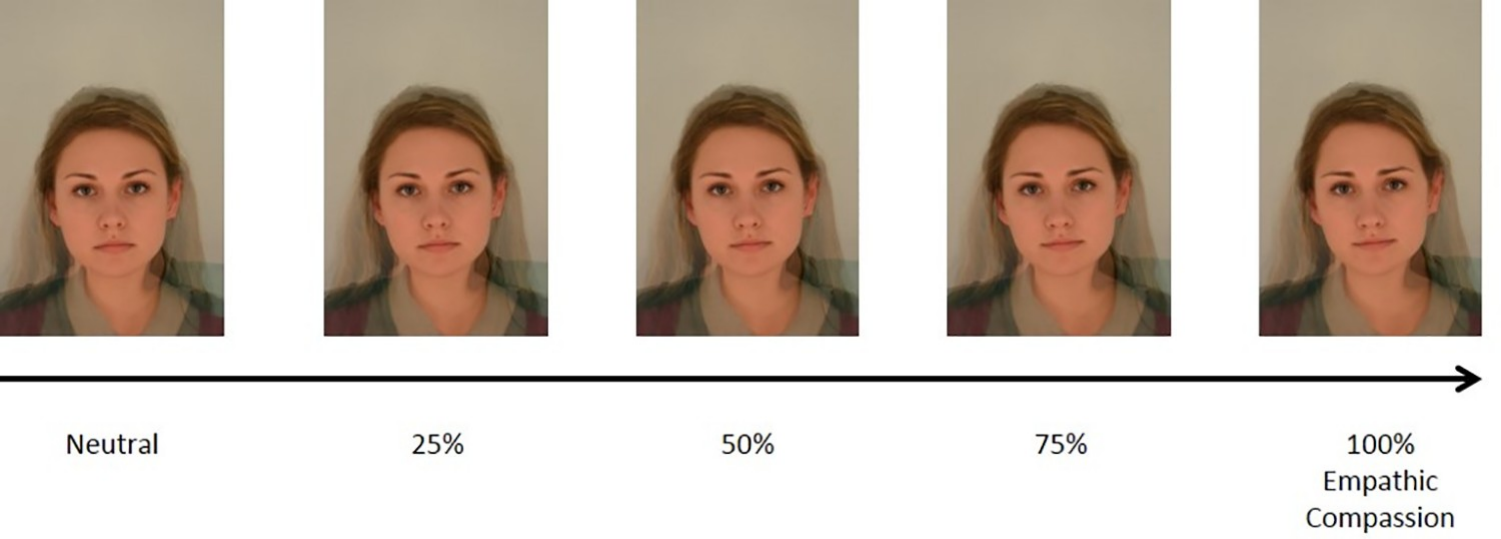
Example of a neutral composite morphed towards the empathic compassion prototype at 25%, 50%, 75% and 100%.

The benefit of using computer graphics software such as PsychoMorph is that new face stimuli can be generated from composite images. This software allows facial features from any image to be registered with markers and then transformed towards the features delineated in a target image [35]. This process, described in more detail under Study 3 methods, was used to generate six face stimuli as exemplars of each type of compassion (an example is provided in the rightmost panel of Fig 2) that we used to investigate the perception of compassion in the following paradigm. We generated the stimuli from the photographs taken in Study 1. The neutral expression photographs from eighteen actors in Study 1 were selected, based on having the lowest emotion ratings, and were randomly divided into groups of three. Six neutral identities were generated by averaging the 3 neutral photos using Psychomorph. Averaging of the neutral faces ensured that the resulting images were not of identifiable individuals. Each of the six neutral identities was then shape-transformed using the differences between the corresponding landmark points of the neutral identity and each of the two compassion composites. Specifically, the coordinates of the landmark points of the neutral face were shifted towards the respective coordinates of the compassion composites. By doing so, 100% of the linear difference in 2D shape between the corresponding landmark points of the neutral stimulus and the compassionate composite were added to the neutral face. Thus, a series of twelve new exemplars of kind and empathic compassion were created (six identities both expressing kind and empathic compassion). Note that the stimuli differed only in facial structure, but not in any other dimension, such as colour or luminance.

Participants were required to decide between one exemplar of kind compassion and one exemplar of empathic compassion that were presented side-by-side. These exemplars were presented in three blocks with their own set of instructions that reflected the different compassion intentions. For each image, participants were asked the question “Which of the two photos best depicts the following description?” Block A had the following description, which corresponds to the general definition of compassion: “Compassion is described as a sensitivity to the suffering of the self and others with a desire and commitment to try to prevent and alleviate that suffering”. In Block B, participants had the following description, which reflects the instructions of the kind compassion imagery task: “The model is trying to convey their understanding of someone’s upsetting situation. They were asked to convey kindness and gentleness towards this other person so as to reassure and soothe them”. Participants in Block C had the following description, which reflects the instructions from the empathic compassion imagery task: “The model is trying to convey their understanding of someone’s emotional pain and their shared suffering. They were asked to convey deep concern, sympathy, kindness and gentleness towards this other person so as to reassure and soothe them”. These descriptions were used to assess whether the imagery instructions used to help participants express compassion lead to discernible differences in facial expressions. Each of the six image pairs (plus the prototypes from Study 1) were presented three times resulting in 21 trials in each block. The presentation of the image location in the pairs (i.e. whether an image appears on the left or right) was randomised. All trials within a block and the order of the blocks were also randomised.

In the second part of Study 2 (n = 53), participants were required to rate the two compassion composites generated in Study 1 on a 7-point Likert scale (1 = “Not at all”; 7 = "Very much so”) for different kinds of expression, including happy, sad, angry, fearful, disgust, surprise, compassion, kindness, concern, and reassurance. Each prototype image was presented separately. All images in this study were presented via the online platform Qualtrics and participants were instructed to set their home computer screens to a resolution of 1280x1024 prior to starting the study.

### Results and discussion

For Block A, participants perceived the faces exemplifying empathic compassion as better depicting the general compassion definition in 59% of their trials. This was significantly different from chance, t (53) = -5.60, p < 0.001. In Block B, participants perceived the kind compassion face as better depicting the kind compassion instructions at approximately chance level (49% of their trials), t (53) = .76, p = .45. Finally, participants in Block C perceived the empathic compassion faces as better depicting the empathic compassion instructions in 62% of their trials. This was significantly different from chance, t (53) = -17.7, p < 0.001.

Paired t-tests revealed significant differences between the two compassion prototypes along several affective dimensions. As in Study 1, Bonferroni corrections would have resulted in setting alpha at .005 and were judged to be inappropriate. The kind compassion face was rated significantly higher for expressions of happiness, t (48) = 10.70, p < 0.001, and kindness, t (48) = 3.5, p = 0.001. The empathic compassion face was rated significantly higher for expressions of sadness, t (48) = 9.64, p < 0.001, anger, t (48) = 2.67, p = 0.01, fear, t (48) = 3.46, p = 0.001, disgust, t (48) = 2.37, p = 0.02, and concern, t (48) = 7.35, p < 0.001. Ratings of reassurance, compassion and surprise were not statistically different (p > .24). A summary of the mean ratings is presented in Table 3.

**Table 3:** Mean (SD) of emotions associated with kind and empathic compassion prototypes.

	Mean (SD) Expressivity Rating									
	Happiness	Sadness	Anger	Fear	Disgust	Surprise	Compassion	Kindness	Concern	Reassurance
Kind Compassion	5.08** (.90)	2.16 (1.10)	1.18 (.43)	1.53 (.90)	1.16 (.42)	1.37 (.75)	4.84 (1.49)	5.86** (1.13)	3.39 (1.61)	4.73 (1.40)
Empathic Compassion	2.98 (1.13)	4.29** (1.27)	1.49* (.92)	2.24** (1.49)	1.41* (.80)	1.29 (.64)	4.96 (1.43)	5.16 (1.17)	5.24** (1.58)	4.41 (1.56)

NB

* = rated significantly higher than the other prototype (p < .05)

** = rated significantly higher than the other prototype (p < .001).

The main finding from this study is that the two compassion prototypes are perceived differently. The kind compassion faces were perceived as kinder and happier than the empathic compassion faces. Compassion is not generally associated with expressions of happiness as it would be odd to meet someone’s suffering with happiness. However, research shows that kindness and happiness are associated [42] and that joy can be experienced as a result of compassion [43]. While expressions of kindness were kept constant in the kind and empathic imagery instructions, the additional focus on understanding, sympathy and concern in the empathic compassion condition have clearly altered the expression and the perception of kindness and happiness. This could also indicate that those individuals in the empathic compassion composite were able to detect the distress (e.g. fear and sadness) in the person they recalled during the induction task and appropriately display marked mirroring.

Of interest are the higher ratings of sadness and concern for the empathic compassion faces. This is consistent with the instructions used in the empathic imagery condition but also with previous findings of facial displays of sympathy that show emotional resonance [19, 20]. In these studies, two actors were asked to express “sympathy” (although the exact instructions to the actor are unclear), which was then interchanged with the label “compassion”. It is not clear from these studies what the motivation of the actor is or whether their facial expression represents vicarious sympathy (e.g. Eisenberg et al., 1988; 1989; 1998). Nevertheless, within this small sample of sympathy face stimuli the results indicated emotional resonance through labels of sadness.

The forced-choice task revealed that the two compassion imagery instructions used to help participants express compassion produced discernible expressions attributable to those instructions. When presented with a description from the empathic imagery task participants consistently chose the empathic compassion faces. For the kind compassion instructions participants selected, at chance, the kind-compassion face. This might reflect the fact that kindness is also present in the empathic faces, as per the instructions. Interestingly, when participants were presented with the general definition of compassion they reliably chose the empathic faces as better representing this definition. Whether there are discernible differences between the two faces is further explored in Study 3.

## Study 3

### Methods

#### Participants

Thirty-four participants (16 females; mean age 20.3, SD = 1.52) participated in this study. Participants had not previously participated in Studies 1 or 2. Formal power analysis for the within-subject analyses was precluded by the absence of information about the likely correlation between the observations. Sample size was therefore gauged on previous literature in the field, with sample sizes ranging from 24 to 50 [29, 30]. The results suggested it was adequate.

#### Procedure

To further validate the faces and investigate differences in the perception of kind and empathic compassion we conducted a discrimination task. This type of paradigm has previously been used to test the perception of subtle changes in faces expressing or possessing certain emotions or qualities along a continuum [29–31, 41].

The stimuli used in the discrimination task were generated from the six neutral composites generated in Study 2. Using a similar process to Study 2, each of the six neutral composites were shape-transformed using the differences between the corresponding landmark points of the neutral composite and the compassion prototypes. Specifically, the coordinates of the landmark points of the neutral face were shifted towards the respective coordinates of the compassion prototype. By doing so, 25%, 50%, 75% and 100% of the linear difference in 2D shape between the corresponding landmark points of the neutral stimulus and the compassionate prototype were added to the neutral face. Thus, a series of five images (for each of the six new identities) ranging from neutral to kind and empathic compassion (0%, 25%, 50%, 75% and 100% compassionate) were created. Fig 2 shows an example of a neutral composite morphed towards the empathic compassion prototype. Note that the transformation was shape transformation only, resulting in stimuli that differed only in facial structure, but not in any other dimension, such as colour or luminance.

In the computer-based discrimination task there were 264 trials that required participants to discriminate which photo, from a pair (of the same person), expressed more compassion. The photos differed from each other by a percentage of transformation along the compassion continuum. There were five levels of transformation along the kind and empathic compassion continuum and the pairings used are in parentheses: 100% (0% vs 100%), 75% (0% vs 75%, 25% vs 100%), 50% (0% vs 50%, 25% vs 75%, 50% vs 100%), 25% (0% vs 25%, 25% vs 50%, 50% vs 75%, 75% vs 100%) and 0% (identical images). The 0% difference condition was included only to balance the design, and was not used for analysis. There were four repetitions of each percentage difference for each of the 6 identities. The position on the screen of the face that was further along the compassion continuum was counterbalanced across the four repetitions.

The discrimination task was programmed using PsychoPy software (Version 1.77.01) [44]. Participants were given the general definition of compassion prior to starting the discrimination task and were presented with onscreen instructions. The experiment proper was preceded by six practice trials. All trials were randomized and were organised into kind and empathic compassion blocks, the order of which were counterbalanced across participants. Participants had to choose the face that they perceived as most compassionate by pressing ‘f’ on the keyboard if it was the face on the left and ‘j’ if it was the face on the right. A central fixation cross was programmed to appear between trials. Participants’ responses and reaction times were recorded. The percentage of correct responses was analysed.

### Results and discussion

**Fig 3:**
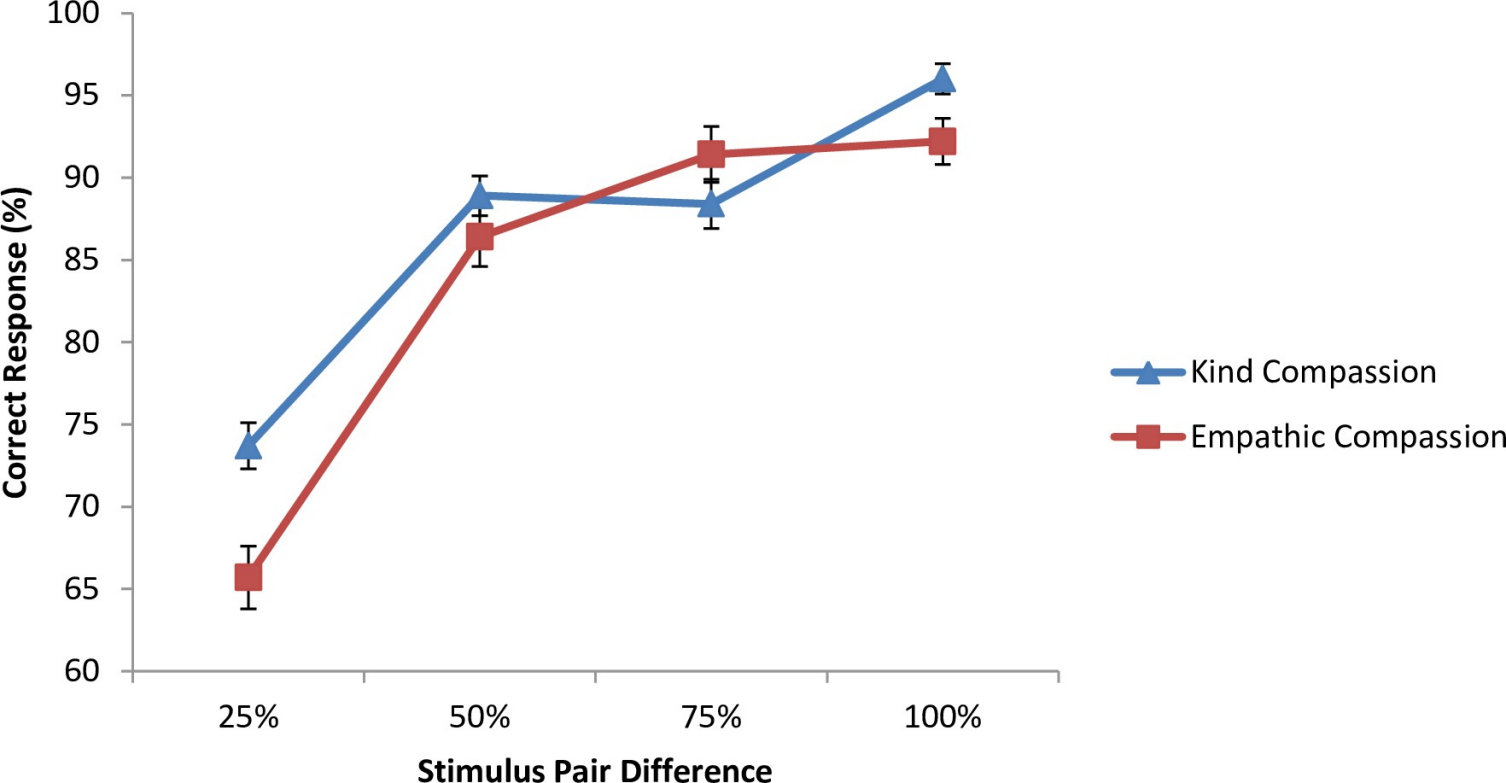
Mean accuracy of kind and empathic compassion discriminations as a function of the percentage of difference between two pairs of images. Error bars represent standard error of the mean.

A 2 (compassion expression) x 4 (pair difference) repeated-measures Analysis of Variance (ANOVA) revealed an overall main effect of compassion expression, F (1, 33) = 6.29, p = .02, partial eta squared = .16. Correct responses for kind compassion faces (M = 89.9%, SE = 1.31) were significantly higher than empathic compassion faces (M = 86.7%, SE = 1.05). There was a significant main effect of pair difference, F (3, 99) = 168.2, p < .001, partial eta squared = .84. Pairwise comparisons across pair difference revealed a statistically significant increase in the percentage of correct responses as the percentage of morph difference between the stimulus pairs increased. Specifically, correct response for a 100% difference between the pairs of images was greater than those with a 25% difference (largest p = .028). Finally, there was a significant interaction between the compassion condition and the percentage of pair difference, F (3, 99) = 9.73, p < .001, partial eta squared = .23. Pairwise comparisons revealed that the kind and empathic faces were significantly different from each other at 25% pair difference (mean difference (SE): 7.97% (1.9); p < .001) and 100% pair difference (mean difference (SE): 3.8% (1.35); p = .01) but not at 50%, or 75% (p > .05). However, t-tests showed that participants' correct responses at each pair difference were above chance for both compassion expressions (p < .001). The findings are graphed in Fig 3.

This study showed that participants were more sensitive to small variations of expressions of kind-compassion than empathic compassion. This was especially the case when stimulus pairs were morphed in such a way as to display a difference of 25% and 100% compassion. That is, it was easier to detect kind compassion when the comparisons between two face stimuli were most obvious (i.e. 100%) and most difficult (i.e. 25%). Importantly, the correct responses for both kind and empathic faces were reliably above chance and increased as a function of the percentage of difference between face pairs, indicating that our morphing manipulation was successful. This result is not surprising as it reflects that participants could more reliably discriminate the images as differences between the two stimuli increased.

The results demonstrate that there is a difference in the way that these two compassionate faces are processed. While the empathic compassionate faces were chosen as more representative of the definition of compassion in Study 2, different intensities of kind compassion were better discriminated. Given that kind compassion faces were rated higher for happiness in Study 2, it could be the case that participants are using this dimension to assist in their discrimination, namely the smile. There is evidence that a smile is processed faster than other features of the face, presumably with the intention to speed up the categorization of the expression for social interaction [45]. Conversely, the complexity of the empathic compassion faces could introduce greater uncertainty when discriminating. Furthermore, we believe that the empathic compassion expression centres around the eyes and eyebrows, and eyes are processed second in the presence of a smile [45].

## General discussion

This study investigated the typology of facial expressions of compassion and how these are perceived by others. In Study 1 we induced either kind- or empathic-compassion facial expressions through an experiential imagery technique, instructing participants to express feelings and their compassionate motivations through facial expressions. Composite images of the most highly rated kind- and empathic-compassion expressions were then generated using computer graphics software. In Study 2 we found perceived differences between the two compassionate expressions in so far as participants attributed the correct corresponding compassion motivation to the empathic-compassion face and consistently associated the empathic-compassion face with the general definition of compassion. In Study 3 participants could reliably discriminate face stimuli along both kind- and empathic-compassion continua, with degrees of kind-compassion being easier to discriminate.

While our composite compassion images did not differ in terms of compassion ratings, the kind-compassion face was rated higher for happiness and kindness. These ratings are consistent with findings from McEwan et al. [21] and further highlight that a soft-smiling, affiliative compassionate expression may be construed as non-empathic or unsuitable in some circumstances of distress. Conversely, the empathic composite was rated higher for sadness, fear and concern. This compassion-sadness 'confusion' [10] is consistent with emotional resonance occurring as part of the identification of suffering in another person and appropriate marked mirroring to convey understanding. Previous research on facial displays of sympathy has also highlighted the presence of sadness or distress [11, 13–15]. If people become distressed by the suffering of others they may try to cut off from that distress (e.g. break contact or dissociate), failing to behave compassionately as a result [4, 11, 13–15]. Compassion appears to require a level of distress tolerance [46]. More broadly, research linking sensitivity to facial expressions of distress with prosocial behaviour [17, 18, 47] may benefit from taking into account distress tolerance as a mediating variable.

Our research adds to existing knowledge by indicating that there is no one specific face of compassion, rather our expressions depend upon context and motivation. The facial expression of someone who is portraying themselves as a safe, friendly listener and who can generally be trusted with personal disclosures may be different to the compassionate face of someone who is actively listening to a story of pain and suffering and seeking to comfort that person. Compassionate facial expressions when we are with an angry person or when we rush in to help someone in danger may also be very different. This highlights again that compassion is a motivation or intention and its facial expressions will depend upon the context in which it is expressed. While the overall motivation behind these two expressions is to engage with suffering and communicate that engagement, they can be seen as having different communicative functions. Adding to the original work of McEwan et al. [21], who identified one compassionate expression, our work suggests that two distinct compassionate facial expressions (rather than one) may better represent the complex cognitive, emotional and behavioural processes of compassion.

### Limitations

Study 1 resulted in a stimulus set of only female faces from a predominantly white Caucasian background, which does not allow us to generalise our findings of compassionate expressions to men or other ethnic groups. Given our initial pilot work with men further research is required to explore methodologies that could assist in capturing compassionate expressions from men and their subsequent perception. We suggest that the expression of compassion is not invariant and by extension it could vary between genders. Men report higher levels of self-compassion compared to women [48] but in the case of compassion for others, women report higher levels than men [49, 50]. This may be due to social norms denoting women’s care-giving and nurturance roles while men are seen to be remote but stable or constant in the face of distress. Sousa et al [50] found that men report higher levels of disconnectedness from others’ suffering including a separation and disengagement from others. Considering this, it may be that men express compassion differently, which may also depend on whether the object of compassion is male or female.

Our photos were also of young adults. There is little research investigating age related changes in the expression and perception of compassion. Older adolescence appears to alter levels of self-compassion in women but not men [48]. The earlier work by Eisenberg et al. [11, 13, 15] would suggest age related differences in the expression of sympathy in young children and adults. In older adults, research has shown a reduction in the accuracy of facial expression recognition, which is associated with perturbed social interaction and ultimately a reduced quality of life [51, 52]. It is possible that, over a lifespan, individuals gain insight and skills from personal experiences and, as a result, older adults may be more confident and better equipped to express compassion.

Interpersonal interactions are, of course, complex and dynamic processes. Our studies used static snapshots of the dynamic situations; future studies should aim towards capturing and quantifying animated compassionate interactions to establish how these evolve across a situation. Compassion also manifests itself beyond facial expressions and future studies should investigate the role of, for example, body posture and tone of voice [53] or approach and avoidance behaviour. Kaltwasser et al. (2017) found that dynamic expressions of fear and sadness elicited more approach responses relative to angry faces, with a larger difference between fear and anger responses in prosocial compared to individualistic participants. This suggests that the social meaning of these expressions (i.e. distress) is a motivational factor in their approach behaviour, which could be used as a proxy for compassion if the intention was to soothe or comfort the individual. A similar approach-avoidance paradigm could also be used to with our stimuli. We would predict compassionate expressions triggering approach behaviour in those experiencing distress. However, this again would depend on the individual beliefs or fears of receiving compassion from others (e.g. “I don’t deserve compassion” or “They’ll see me as weak if I come to them”) [33].

## Conclusions

The key finding from the current research is the generation of two perceptually and functionally distinct facial expressions of compassion. This finding contributes to the field not only by providing insight into the expression and perception of compassion but also highlights differences in the way that compassion is operationalised. The compassionate composites have the potential to generate a stimulus set that can also be used to address the gap in the literature more generally around the perception of complex, social facial expressions.

## Supporting information

S1 Dataset Data for Studies 1–3. (SAV)
